# Clinical and Dietary Determinants of Muscle Mass in Patients with Type 2 Diabetes: Data from the Diabetes and Lifestyle Cohort Twente

**DOI:** 10.3390/jcm10225227

**Published:** 2021-11-10

**Authors:** Annis C. Jalving, Milou M. Oosterwijk, Ilse J. M. Hagedoorn, Gerjan Navis, Stephan J. L. Bakker, Gozewijn D. Laverman

**Affiliations:** 1 Department of Internal Medicine/Nephrology, ZGT Hospital, 7609 PP Almelo, The Netherlands; mi.oosterwijk@zgt.nl (M.M.O.); ilse_hagedoorn10@hotmail.com (I.J.M.H.); g.laverman@zgt.nl (G.D.L.); 2 Department of Internal Medicine, Division of Nephrology, University of Groningen, University Medical Center Groningen, 9713 GZ Groningen, The Netherlands; g.j.navis@umcg.nl (G.N.); s.j.l.bakker@umcg.nl (S.J.L.B.); 3 Faculty of Electrical Engineering, Mathematics and Computer Science (EEMCS) and Biomedical Signals and Systems (BSS), University of Twente, 7522 NB Enschede, The Netherlands

**Keywords:** diabetes type 2, lifestyle, muscle mass, cohort study

## Abstract

Low muscle mass in patients with type 2 diabetes is associated with a progressively higher risk of morbidity and mortality. The aim of this study was to identify modifiable targets for intervention of muscle mass in type 2 diabetes. Cross-sectional analyses were performed in 375 patients of the Diabetes and Lifestyle Cohort Twente-1 study. Muscle mass was estimated by 24 h urinary creatinine excretion rate (CER, mmol/24 h). Patients were divided in sex-stratified tertiles of CER. To study determinants of CER, multivariable linear regression analyses were performed. Protein intake was determined by Maroni formula and by a semi-quantitative Food Frequency Questionnaire. The mean CER was 16.1 ± 4.8 mmol/24 h and 10.9 ± 2.9 mmol/24 h in men and women, respectively. Lower CER was significantly associated with older age (*p* < 0.001) as a non-modifiable risk factor, whereas higher BMI (*p* = 0.015) and lower dietary protein intake (both methods *p* < 0.001) were identified as modifiable risk factors for lower CER. Overall body mass index (BMI) was high, even in the lowest CER tertile the mean BMI was 30.9 kg/m^2^, mainly driven by someone’s body weight (*p* = 0.004) instead of someone’s height (*p* = 0.58). In the total population, 28% did not achieve adequate protein intake of >0.8 g/kg/day, with the highest percentage in the lowest CER tertile (52%, *p* < 0.001). Among patients with type 2 diabetes treated in secondary care, higher BMI and low dietary protein intake are modifiable risk factors for lower muscle mass. Considering the risk associated with low muscle mass, intervention may be useful. To that purpose, dietary protein intake and BMI are potential targets for intervention.

## 1. Introduction

Over the past few decades, the prevalence of type 2 diabetes has dramatically increased in parallel to the obesity epidemic [1,2]. Both are closely related to a sedentary lifestyle and adverse dietary habits [3,4,5]. High body fat percentage, with concomitant insulin resistance, is considered the key abnormality in body composition in type 2 diabetes. However, it is increasingly recognized that low muscle mass per se also carries a considerable risk in type 2 diabetes [6,7]. Muscle cells are important in several metabolic mechanisms, including glucose disposal, which is relevant in maintaining insulin sensitivity [8]. A low muscle mass could easily go unnoticed, especially in obese individuals. Obese adults with low muscle mass have a higher risk of developing frailty and disability and thus poor quality of life [6,9]. Furthermore, low muscle mass is associated with higher mortality in obese patients with chronic diseases, including type 2 diabetes [10]. Of note, the progressively higher risk of mortality with lower muscle mass in type 2 diabetes applies to the full range of muscle mass [10]. Therefore, it is recommended that efforts should be made to prevent or minimize loss of skeletal muscle mass in these patients [6].

The Diabetes and Lifestyle Cohort Twente (DIALECT) study is specifically designed to study lifestyle characteristics, including diet, physical activity scores and outcomes in patients with type 2 diabetes treated in secondary care. The extensive data collection in this study includes collection of 24 h urine samples, providing a method for estimating muscle mass by determining 24 h creatinine excretion rate [11]. The aim of the present study was to identify potentially modifiable determinants of muscle mass, including nutrition and physical activity, in this population of patients with type 2 diabetes.

## 2. Materials and Methods

### 2.1. Study Design

This was a cross-sectional study using baseline data from the Diabetes and Lifestyle Cohort Twente-1 (DIALECT-1). DIALECT-1 was performed in the outpatient clinic of the hospital Ziekenhuis Groep Twente (ZGT), Almelo and Hengelo, The Netherlands. The ZGT hospital is a secondary health care center for diabetes treatment. In the Netherlands, criteria for referral from primary to secondary health care are inability to achieve adequate glycemic control with oral blood glucose lowering agents or a standard insulin regimen, macroalbuminuria and/or estimated Glomerular Filtration Rate (eGFR) ≤ 60 mL/min/1.73 m^2^, or multiple cardiovascular complications. The study has been approved by local institutional review boards (METC-Twente, NL57219.044.16; METC-Groningen, 1009.68020), is registered in The Netherlands Trial Register (NTR trial code 5855) and was performed according to the Guidelines of Good Clinical Practice and the Declaration of Helsinki.

### 2.2. Population

The study population and study procedures have been described previously [12,13]. In brief, 433 patients aged 18+ years with type 2 diabetes were included and exclusion criteria were renal replacement therapy or inability to understand the concept of informed consent. For the current study, we excluded patients with missing CER data (*n* = 4), missing subjective data on dietary intake (*n* = 11) and missing objective dietary protein intake (*n* = 43), leaving 375 patients for analysis. A patient inclusion flowchart was shown in Figure S1.

### 2.3. Dietary Assessment

Objective total protein intake (g/day) was determined by the Maroni formula [14]: 6.25 × ((0.0276 × urinary urea excretion (mmol/24 h)) + (0.031 × body weight (kg))) + urinary protein excretion. Protein intake of at least 0.8 g/kg body weight per day is advised by the World Health Organization for healthy adults [15]. For healthy older adults > 65 years an intake of at least in the range of 1.0–1.2 g/kg/day and for older adults who have acute or chronic diseases an intake of 1.2–1.5 g/kg/day is advised [16]. For this reason, we performed an additional analysis in which we categorized total protein intake in four groups: <0.8 g/kg/day, 0.8 to <1.0 g/kg/day, 1.0 to <1.2 g/kg/day, and ≥1.2 g/kg/day. Ideal body weight was used to estimate total protein intake in g/kg/day, based on an arbitrarily optimal BMI of 25 kg/m^2^, corresponding with current nutritional recommendations.

The origin and sources of dietary protein and relative distribution of food components were determined using a semi-quantitative Food Frequency Questionnaire (FFQ), which was previously validated at the Wageningen University and modified for accurate measurement of protein intake [17]. The FFQ contains intake of 177 items during the last month, taking seasonal variations into account. For each item, the frequency was recorded in times per day, week or month. The number of servings was expressed in natural units (e.g., slice of bread or apple) or household measures (e.g., cup or spoon). Intake of macronutrients was calculated using the Dutch Food Composition Table of 2013 [18]. Based on the Dutch Food Composition Table, three categories of proteins were defined, the first category consisting of meat, poultry and fish (animal origin), the second of dairy and eggs (animal origin) and the third category consisting of protein originating from plants, i.e., nuts, vegetables, fruit, etc. Protein intake of several different protein-rich food categories was calculated by adding up protein content across all food items in the specific category.

### 2.4. Outcome Measurement

Muscle mass was estimated by 24 h urinary creatinine excretion rate (CER). Patients were asked to collect their 24 h urine to obtain the urinary CER, by multiplying these concentrations with the volume of the 24 h urine collection. Patients were instructed to store the canister in a dark, cool place, preferably in a refrigerator. CER has become a well-accepted method of measuring muscle mass [11,19], and is based on the rationale that in a steady state creatinine is produced at a constant rate. This directly reflects the quantity of total muscle mass, as creatinine is formed from the non-enzymatic conversion of creatine and creatine phosphate in muscle. Recently there have been studies that suggest that CER not only measures muscle mass, but could also be an indicator of muscle performance [20,21]. 

### 2.5. Modifiable and Non-Modifiable Risk Factors

Non-modifiable risk factors were demographic factors such as sex, age and height (cm). Sociodemographic characteristics and medical history of participants, as well as current medications, were also collected during the study visit. 

Modifiable risk factors consisted of both directly modifiable, i.e., diet and physical activity, and preventable risk factors, i.e., complications of diabetes. Anthropometric dimensions, body weight (kg) and waist and hip circumference (cm), were measured using standard procedures and body mass index (BMI) was calculated as weight divided by squared height (kg/m^2^). Body surface area (BSA) in m^2^ was determined with Dubois and DuBois formula: (weight(kg)^0.425^ × height(cm)^0.725^) × 0.007184 [22]. Physical activity was subjectively measured by the validated Short Questionnaire to Assess Health-enhancing Physical Activity (SQUASH) [23]. Target of physical activity was at least 30 min of moderate–vigorous exercise at least 5 days per week [24]. Additionally, performance of at least any physical exercise on top of normal daily activities was assessed with the SQUASH questionnaire. Routine laboratory tests were performed in non-fasting venous blood, including renal function tests, HbA1c and LDL cholesterol. The eGFR was calculated using the Chronic Kidney Disease Epidemiology Collaboration (CKD-EPI) formula [25]. Nephropathy was defined as either microalbuminuria (24 h albumin excretion of 30–300 mg/day) or macroalbuminuria (24 h albumin excretion of >300 mg/day), or renal function impairment (eGFR < 60 mL/min/1.73 m^2^) [26]. Peripheral artery disease was defined as atherosclerotic peripheral vessel disease, or aneurism of the aorta. Coronary heart disease was defined as physician-diagnosed unstable angina pectoris or myocardial infarction, percutaneous coronary intervention or a coronary artery bypass graft in the medical history [27].

### 2.6. Statistics 

All statistical analyses were performed using SPSS version 23.0 (IBM, Chicago, IL, USA). Normality of data was assessed by visual inspection of frequency histograms. Normally distributed variables were presented as mean ± standard deviation, skewed variables as median (interquartile range) and dichotomous variables as numbers (percentage). A two-tailed *p*-value < 0.05 was considered statistically significant.

Patients were divided in sex-stratified CER tertiles to group them in categories of muscle mass, as there are no specific cut-off values for normal range of muscle mass measured by CER so far. Differences between groups were tested using one-way ANOVA test (normal distribution), Kruskal–Wallis test (skewed distribution) and Chi-Square (categorical). Differences between men and women were tested by using an independent *t*-test.

To study the determinants of CER in patients with type 2 diabetes in our cohort, multivariable linear regression analyses were performed. All univariable associations with a *p*-value < 0.20 and variables that were found to be associated with muscle mass were included in a forward linear regression model (standardized beta, *p*-value) with CER as the dependent variable [6]. Model 1 was adjusted for age and sex, model 2 was additionally adjusted for height and weight, model 3 was additionally adjusted for dietary protein intake according to the Maroni formula (g/day), model 4 was additionally adjusted for physical exercise. 

### 2.7. Sensitivity Analysis 

Multiple sensitivity analyses were performed. First, we excluded subjects with potential under- or overcollection from analysis. Such samples were identified through assessing the difference between the estimated and measured volume of a subject’s 24 h urine sample. The estimated 24 h urine volume was derived from the formula: Creatinine clearance = ([urine creatinine] × 24 h urine volume)/[serum creatinine], where creatinine clearance was estimated using the Cockcroft–Gault formula [28]. If accurately collected, estimated creatinine clearance using 24 h urine specimens and estimated creatinine clearance using a serum-based equation (which is entirely independent of the timed urine) should be similar. Samples with potential under- or overcollection were defined as the upper and lower 2.5% of the difference between the estimated and measured volume of a subject’s 24 h urine sample. Second, we excluded patients using either trimethoprim or cimetidine, drugs which could influence tubular creatinine secretion. Third, we performed analyses in which we repeated multivariable linear regression analysis with either CER divided by BSA or with Skeletal Muscle Index (SMI, kg/m^2^) as main outcome in the fully adjusted model. SMI was calculated as muscle mass (kg) divided by squared height (kg/m^2^). Muscle mass (kg) was calculated with the formula: [18.9 × (CER (mmol/24 h)/8.84)] + 4.1 [29]. Finally, we repeated multivariable linear regression analysis with urinary urea excretion (mmol/24 h) in the fully adjusted model instead of dietary protein intake according to the Maroni formula.

## 3. Results

Baseline characteristics by sex-stratified tertiles of CER are shown in Table 1. Female patients have a lower mean CER than males (10.9 ± 2.9 mmol/24 h versus 16.1 ± 4.8 mmol/24 h resp.; *p* < 0.001, data not shown) and CER was lower with increasing age. As expected, higher CER and height were correlated (*p* = 0.002). Additionally, both total urinary volume (*p* < 0.001) and urinary creatinine concentration (*p* < 0.001) were higher across tertiles of CER. 

Although body mass index was higher with stepwise increasing CER, even patients in the lowest CER tertile were still obese with a mean BMI of 30.9 kg/m^2^. Other body dimensions, such as waist circumference and BSA, were also higher across tertiles of CER. CER was positively associated with dietary protein intake, either determined from the Maroni formula or from the FFQ. The percentage of patients achieving the target for physical activity was not significantly different across CER tertiles (*p* = 0.20), but the percentage of patients performing at least any physical exercise on top of normal daily activities was higher in the highest tertile of muscle mass compared to the lowest tertile of muscle mass (*p* = 0.023). Urea excretion was significantly higher in the highest tertile of CER (313 ± 107 vs. 408 ± 113 vs. 529 ± 141 mmol/24 h, *p* < 0.001). There was a slight difference in results from our analyses on protein intake from the different sources: total protein intake determined by Maroni formula was 91 g/day, while total protein intake determined by the FFQ was 80 g/day. Scatterplots with urinary creatinine excretion on the *x*-axis and dietary protein intake in gram/day and gram/kg/day on the *y*-axis, stratified by sex, are shown in Figure S2.

### 3.1. Determinants of CER

Multivariable linear regression analysis with CER as the dependent variable identified multiple prognostic risk factors. The crude model, with age and sex as non-modifiable prognostic risk factors, contributed to 34.5% of the variance in CER (*p* < 0.001) (Table 2). Height and weight had an incremental 8.2% of variance, and adding Maroni-based protein intake to the regression analysis, the adjusted R2 increased to 71.4%. Physical exercise did not additionally increase the variance, but the model remained significant. Maroni-based protein intake contributed independently to CER after controlling for the effects of age, sex, height, weight and physical exercise (Std. Beta = 0.640, *p* < 0.001). 

### 3.2. Additional Analyses on Protein Intake 

**Figure 1:**
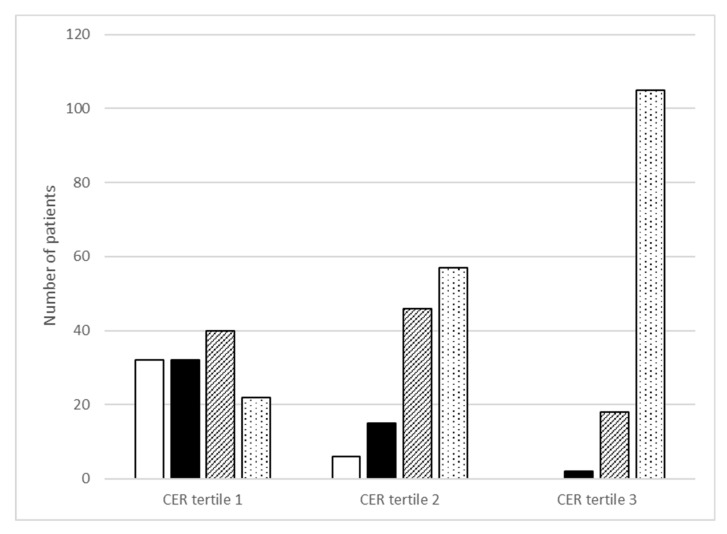
Categories of Maroni-based protein intake (g/kg/day) across sex-stratified tertiles of creatinine excretion rate (CER). White bar is protein intake < 0.8 g/kg/day (*n* = 32; *n* = 6, *n* = 0); black bar is 0.8 to <1.0 g/kg/day (*n* = 32, *n* = 15, *n* = 2); striped bar is 1.0 to <1.2 g/kg/day (*n* = 40, *n* = 46, *n* = 18) and white bar with dots is ≥1.2 g/kg/day (*n* = 22, *n* = 57, *n* = 105). Differences across CER tertiles were statistically significant for each protein category (*p* < 0.001).

Figure 1 shows the distribution of categories of total protein intake across the tertiles of CER. Overall, in almost half of the patients the protein intake was above 1.2 g/kg/day (49%). Regarding tertiles of CER, only 18% of the patients in the lowest tertile consume this amount of dietary protein per day, compared to 46 and 84% in the second and third tertile of CER, respectively (*p* < 0.001). On the other hand, 25% of patients in the lowest tertile did not achieve the recommended intake of protein of >0.8 g/kg/day, compared to 5 and 0% in the second and third tertile of CER, respectively (*p* < 0.001). 

Table 3 shows sources of protein intake. Differences between the CER groups appear to be driven by differences in animal protein intake, being the only source of protein significantly associated with CER (*p* = 0.011). In the food group analyses, we found that the three main groups (i.e., animal origin meat, animal origin diary and eggs and plant based) of dietary sources for protein contributed almost equally to total protein intake. Protein from animal origin, in terms of meat, poultry and fish, was significantly and positively associated with increasing tertiles of CER (*p* = 0.002). Animal-based protein originating from dairy and eggs and plant-based protein were not associated with CER (*p* = 0.33 and 0.66, respectively).

#### Sensitivity Analysis

We performed several sensitivity analyses. In the analyses where we excluded either 24 h urine samples with possible under- or overcollections or patients using drugs which could influence tubular creatinine secretion, there were no material differences compared to the results of the primary analyses. The sensitivity analyses in which we repeated analyses with SMI as main outcome in the fully adjusted model remained materially unchanged compared to the primary analyses. Maroni-based protein intake also remained significant with CER normalized to BSA as the dependent variable. Additionally, urinary urea excretion instead of dietary protein intake according to the Maroni formula contributed independently to CER after controlling for the effects of age, sex, height and weight.

## 4. Discussion

This study evaluated the non-modifiable and modifiable factors of muscle mass in patients with type 2 diabetes by using 24 h urine creatinine excretion rate (CER). The study was performed in the DIALECT-1 population, a referred care cohort, in which extensive phenotyping has been performed in particular with respect to nutrition and physical activity [12,30,31]. The main finding of this study was that low protein intake and high BMI as modifiable risk factors are associated with lower muscle mass in patients with type 2 diabetes.

It is well known that the intake of dietary protein has a positive effect on muscle protein synthesis. Therefore, a dietary protein intake of at least 0.8 g/kg/day is recommended by the World Health Organization for healthy adults [15]. Because of certain anabolic stimuli, obese adults who suffer from chronic diseases may need higher protein intake (1.2–1.5 g/kg/day) compared to lean people to maintain or regain muscle proteins [16]. This would mean that only 18% of patients in the lowest tertile of CER consumes enough protein to regain muscle mass, and that even one-quarter of patients in the lowest tertile of CER did not meet the recommendation for healthy adults. In order to maintain adequate nutritional status and prevent malnutrition, it is of utmost importance to focus on an adequate protein intake in patients with type 2 diabetes. These facts are remarkable since dietary protein intake was higher in our study population compared with the general Dutch population (1.26 vs. 1.10 g/kg/day in men, 1.16 vs. 0.96 g/kg/day in women, respectively) [32]. This might reflect diet adaptations, since a low-carbohydrate diet is recommended for patients with type 2 diabetes in order to manage glycemic control and to prevent complications [5,33]. In addition, dietary protein restriction is only limited to patients with advanced renal disease, i.e., patients with eGFR < 30 mL/min/1.73 m^2^ [34]. Since only 3% of our population have an eGFR < 30 mL/min/1.73 m^2^, it is unlikely that this affects the high percentage of patients with inadequate protein intake.

A paramount finding is that patients in the lowest tertile for muscle mass still have a high mean BMI of 31 kg/m^2^ and lack apparent clinical characteristics to provide a straight-forward diagnosis of sarcopenia. The problem of detecting sarcopenia in obese patients has previously been addressed [6]. A lower muscle mass in type 2 diabetes will almost certainly remain unrecognized in clinical practice unless specific efforts are undertaken to this end. We have recently demonstrated in this population that, in retrospect, their BMI had not changed in a timespan of two decades [35]. Together with the relation between age and CER in the current cross-sectional study, this finding is consistent with the notion that a stable BMI masks a gradual replacement of muscle mass by fat tissue. 

Regarding diet composition, patients in the lowest tertile of CER also had the lowest dietary energy intake and lowest intake of the macronutrients carbohydrates and fat based on the FFQ data. Literature shows that hypocaloric diets, without proper balance in macronutrients, lead not only to loss of fat mass, but often also to a loss of muscle mass, presuming that diet composition is more important than caloric quantity [35]. In fact, it is estimated that with hypocaloric diets about 25% of weight loss is actually skeletal muscle mass in obese older adults [36]. With respect to the origin and source of protein, it is presumed that the intake of dietary protein originating from animals improves muscle mass [36,37]. This is mainly because animal-based protein sources provide certain essential amino acids and tend to be more anabolic. On the other hand, in the highest tertile of CER, whereas the intake of protein is appropriately higher, the dietary energy intake and BMI are, too. In these patients the dietary energy restriction should imply to cut back on dietary carbohydrates and fat intake, thus maintaining high protein intake, and their activity should be increased to lower their BMI [36]. 

Many factors may have an impact on muscle mass, among which are growth hormone, IGF-1, testosterone or thyroid hormones [38,39]. Unfortunately, data regarding growth hormone, IGF-1 and testosterone were not available. In our study population, however, there was no relation between thyroid function (serum T4 and TSH) and muscle mass. Physical activity may also be an important modifiable factor in either increasing muscle mass or lower body weight. However, performance of at least any physical exercise on top of normal daily activities was not an independent determinant of muscle mass. Of note, it has been previously demonstrated that the self-reported physical activity as applied here provides an overestimation [40]. Unfortunately, we were not able to draw conclusions about physical activity since no functional data on muscle performance are available. However, previous DIALECT findings showed that low physical activity (i.e., <5000 steps/day) was associated with higher BMI, lower CER and lower dietary protein intake [41]. 

In prior studies we have shown an inverse association between higher risk of mortality and lower muscle mass over the full range of CER in type 2 diabetes [10], therefore, we chose not to use a lower cut-off for CER in the current study. Of note, the patients in the lowest tertile clearly have a lower CER than previously found in the general population in our country [42], and even compared with kidney transplant patients [20], i.e., a group notoriously known to have decreased muscle mass (10 ± 2.5 vs. 13 ± 4.1 vs. 11 ± 4.0, respectively). 

The main strengths of this study are the availability of data on muscle mass in the real-world setting, together with extensive dietary data using a FFQ that was specifically designed to evaluate protein intake and sources of protein intake. Although the FFQ may not be valid for assessment of absolute dietary protein intake, it is still a valid method to rank individuals according to their intake. For quantitative dietary protein intake, we were able to make use of objective protein intake as calculated by the Maroni formula. 

Limitations of this study are that no cause-and-effect relationships can be proven due to the cross-sectional setting and the fact that the presence of comorbid conditions such as fatty liver or liver cirrhosis which can theoretically affect urinary creatinine levels were not taken into account. Although evaluation of muscle mass based on 24 h creatinine excretion rate has been validated as a measure of muscle mass [11], 24 h urine collection is not available in all settings and is at risk of collection faults. Additionally, day-to-day variation in diet can explain some of the variation in creatinine excretion, but substantial effects on creatinine excretion can only be observed if, after prolonged low protein creatine-free (meat-free) diets, endogenous creatine pools start to decrease, resulting in a decrease in endogenous creatinine generation [43]. In addition, because BMI and lower dietary protein intake were identified as potentially modifiable risk factors for lower muscle mass in patients with type 2 diabetes, it would have been interesting if we could also have investigated a control population without type 2 diabetes. Unfortunately, we did not include a control population without type 2 diabetes in the current study, rendering it not possible to perform such a comparison.

Whereas type 2 diabetes is traditionally considered a disease of abundance, our study re-emphasizes that the high BMI in these patients may well mask the coexistent loss of muscle mass. Hence, low muscle mass may be overlooked as a target for intervention. Muscle mass is modifiable and thereby potentially a target to improve morbidity and mortality in patients with type 2 diabetes. Future studies should investigate whether interventions aimed to improve muscle mass may improve long-term outcome in patients with type 2 diabetes. To that purpose, diet quality should be a main issue, as it can target both low muscle mass and high BMI. A low carb–high protein dietary intervention would be a promising approach. Apart from the need for interventions, systematic documentation of diet, including protein intake, and objective measurements of physical activity may provide a basis to improve lifestyle management in clinical practice [38]. 

## Figures and Tables

**Table 1:** Baseline patient characteristics with sex-stratified tertiles of creatinine excretion rate.

Variable	Total Population	Tertile 1	Tertile 2	Tertile 3	*p*-Value
	*n* = 375	*n* = 126	*n* = 124	*n* = 125	
CER *_a_*, 1, mmol/24 h	13.9 ± 4.9	9.9 ± 2.6	13.7 ± 2.6	18.3 ± 4.7	<0.001
CER *_a_*, 1 females, mmol/24 h	10.9 ± 2.9	7.8 ± 1.6	10.9 ± 0.6	14.0 ± 1.9	<0.001
CER *_a_*, 1 males, mmol/24 h	16.1 ± 4.8	11.3 ± 2.1	15.7 ± 1.1	21.3 ± 3.6	<0.001
Sex, female (%)	158 (42)	53 (42)	53 (43)	52 (42)	0.98
Age, years	63 ± 9	66 ± 9	64 ± 8	60 ± 8	<0.001
Diabetes duration, years	11 (7–18)	11 (7–18)	12 (7–18)	11 (6–18)	0.50
Height, cm	172 ± 10	170 ± 9	172 ± 10	174 ± 9	0.002
Weight, kg	97 ± 18	89 ± 17	95 ± 16	106 ± 18	<0.001
BMI, kg/m^2^	32.7 ± 5.8	30.9 ± 5.3	32.2 ± 5.9	35.0 ± 5.3	<0.001
Waist circumference, cm *	112 ± 13	109 ± 13	110 ± 13	116 ± 13	<0.001
Body surface area, m^2^	2.09 ± 0.22	2.01 ± 0.22	2.08 ± 0.19	2.20 ± 0.21	<0.001
Serum HbA1c, (mmol/mol) *	57 ± 12	56 ± 13	57 ± 11	58 ± 11	0.21
Insulin use, *n* (%)	235 (63)	75 (60)	84 (68)	76 (61)	0.35
Systolic blood pressure, mmHg	139 ± 16	138 ± 17	142 ± 17	138 ± 14	0.047
Diastolic blood pressure, mmHg	76 ± 10	74 ± 10	77 ± 9	77 ± 9	0.025
LDL, mmol/L *	1.99 ± 0.72	2.00 ± 0.72	1.98 ± 0.72	2.00 ± 0.73	0.97
Total urinary volume (mL/24 h)	2025 ± 804	1804 ± 680	2068 ± 820	2208 ± 855	<0.001
Urinary creatinine concentration (mmol/L)	7.6 ± 3.1	6.2 ± 2.5	7.5 ± 2.8	9.0 ± 3.1	<0.001
Urinary urea excretion, mmol/24 h	417 ± 150	313 ± 107	408 ± 113	529 ± 141	<0.001
Microvascular disease, *n* (%)	243 (65)	81 (64)	88 (71)	74 (59)	0.15
Nephropathy, *n* (%)	145 (39)	58 (46)	44 (36)	43 (34)	0.11
eGFR *_b_* < 60, *n* (%)	87 (23)	37 (29)	23 (19)	27 (22)	0.11
Albuminuria, *n* (%)	116 (31)	46 (37)	34 (27)	36 (29)	0.25
Retinopathy, *n* (%)	88 (24)	27 (21)	35 (28)	26 (21)	0.31
Peripheral neuropathy, *n* (%)	136 (36)	46 (37)	43 (35)	47 (38)	0.89
Macrovascular disease, *n* (%)	139 (37)	54 (43)	47 (38)	38 (30)	0.12
Coronary artery disease, *n* (%)	84 (22)	30 (24)	28 (23)	26 (21)	0.85
Cerebrovascular disease, *n* (%)	45 (12)	19 (15)	17 (14)	9 (7)	0.12
Peripheral artery disease, *n* (%)	21 (6)	13 (10)	3 (2)	5 (4)	0.016
Lifestyle parameters					
Target physical activity, *n* (%)	197 (53)	74 (59)	59 (48)	64 (52)	0.20
Physical exercise, *n* (%)	86 (26)	21 (19)	27 (25)	38 (35)	0.023
Current smokers, *n* (%)	64 (17)	24 (19)	19 (15)	21 (17)	0.73
Units of alcohol/month	6 (0–29)	8 (0–32)	5 (0–28)	8 (0–34)	0.31
Dietary intake					
Maroni-based protein intake, g/day	91 ± 27	72 ± 19	89 ± 20	112 ± 25	<0.001
Maroni-based protein intake, g/kg/day	0.95 ± 0.27	0.83 ± 0.26	0.95 ± 0.23	1.07 ± 0.25	<0.001
Adjusted Maroni-based protein intake, g/kg/day *_c_*	1.22 ± 0.32	0.99 ± 0.25	1.20 ± 0.24	1.47 ± 0.28	<0.001
FFQ *_d_* Total energy intake, kCal/day	2010 ± 677	1932 ± 697	1985 ± 596	2112 ± 723	0.10
FFQ Protein, g/day	80 ± 23	76 ± 23	78 ± 21	84 ± 25	0.018
FFQ Protein, en%	16 ± 3	16 ± 3	16 ± 3	17 ± 4	0.58
FFQ Carbohydrates, g/day	207 ± 69	200 ± 72	210 ± 61	212 ± 73	0.34
FFQ Carbohydrates, en%	42 ± 7	42 ± 8	43 ± 6	40 ± 7	0.012
FFQ Fat, g/day	89 ± 40	85 ± 42	86 ± 36	96 ± 40	0.06
FFQ Fat, en%	39 ± 7	38 ± 8	38 ± 6	40 ± 6	0.027

The cut-off points between the tertiles were 13.9 mmol/24 h and 17.7 mmol/24 h for males and 9.9 mmol/24 h and 11.9 mmol/24 h for females. *_a_*: Creatinine Excretion Rate, *_b_*: estimated Glomerular Filtration Rate; *_c_*: adjusted for ideal body weight; *_d_*: Food Frequency Questionnaire * Missing values for waist circumference (*n* = 5), serum HbA1c (*n* = 1), LDL (*n* = 16).

**Table 2:** Creatinine excretion rate/24 h, multiple linear regression. Forward multivariable linear regression analysis with CER as dependent variable (standardized beta, *p*-value). Model 1 is adjusted for age and sex, model 2 is additionally adjusted for height and weight, model 3 is additionally adjusted for dietary protein intake according to the Maroni formula (g/day), model 4 is additionally adjusted for performance of at least any physical exercise on top of normal daily activities.

	Model 1		Model 2		Model 3		Model 4	
	Std. Beta	*p*-Value	Std. Beta	*p*-Value	Std. Beta	*p*-Value	Std. Beta	*p*-Value
Age, years	−0.254	<0.001	−0.138	0.001	−0.129	<0.001	−0.124	<0.001
Sex, female	−0.557	<0.001	−0.430	<0.001	−0.298	<0.001	−0.311	<0.001
Weight, kg			0.275	<0.001	0.096	0.004	0.111	0.002
Height, cm			0.103	0.09	−0.024	0.58	−0.049	0.29
Dietary protein intake (g/day)					0.634	<0.001	0.640	<0.001
Physical exercise, yes							−0.030	0.32

**Table 3:** Origin and sources of protein intake across sex-stratified tertiles of creatinine excretion rate, based on food frequency questionnaire intake.

		Sex-Stratified Tertiles of Creatinine Excretion Rate			
Variable	Total Population	Tertile 1	Tertile 2	Tertile 3	*p*-Value
	*n* = 375	*n* = 126	*n* = 124	*n* = 125	
Total protein intake, g/day	80 ± 23	76 ± 23	78 ± 21	84 ± 25	0.018
Origin of protein					
Plant based, g/day	28 ± 9	27 ± 10	27 ± 8	28 ± 9	0.54
Plant based, en%	5.6 ± 1.2	5.8 ± 1.4	5.6 ± 1.0	5.5 ± 1.1	0.18
Animal based, g/day	52 ± 18	49 ± 18	51 ± 16	56 ± 20	0.011
Animal based, en%	10.8 ± 3.4	10.6 ± 3.2	10.6 ± 2.8	11.1 ± 4.0	0.34
Sources of protein					
Animal origin, meat, poultry, fish, g/day	28 ± 11	27 ± 11	27 ± 11	31 ± 12	0.002
Animal origin, dairy and eggs, g/day	23 ± 13	22 ± 12	23 ± 12	24 ± 15	0.31
Plant origin, g/day	28 ± 9	28 ± 10	28 ± 8	29 ± 10	0.66

The cut-off points between the tertiles were 13.9 mmol/24 h and 17.7 mmol/24 h for males and at 9.9 mmol/24 h and 11.9 mmol/24 h for females.

## Data Availability

The authors will consider every reasonable request to inspect the data used for this article.

## Supplementary Materials

The following are available online at https://www.mdpi.com/article/10.3390/jcm10225227/s1, Figure S1: Patient inclusion flowchart; Figure S2: Scatterplots with urinary creatinine excretion on the *x*-axis and dietary protein intake in gram/day and gram/kg/day on the *y*-axis, stratified by sex.
