# Supplementary material for: Clinical and Dietary Determinants of Muscle Mass in Patients with Type 2 Diabetes: Data from the Diabetes and Lifestyle Cohort Twente
Source: J Clin Med. 2021 Nov 10;10(22):5227. doi: 10.3390/jcm10225227 (PMC8623198; doi:10.3390/jcm10225227)
Supplement: Supplementary file 1 [file jcm-10-05227-s001.zip › jcm-1416659-SI.pdf]

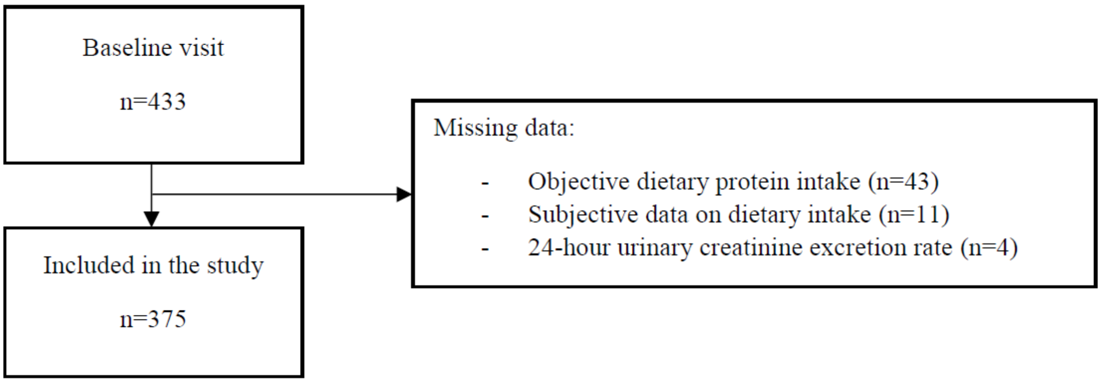

Figure S1. Patient inclusion flowchart.

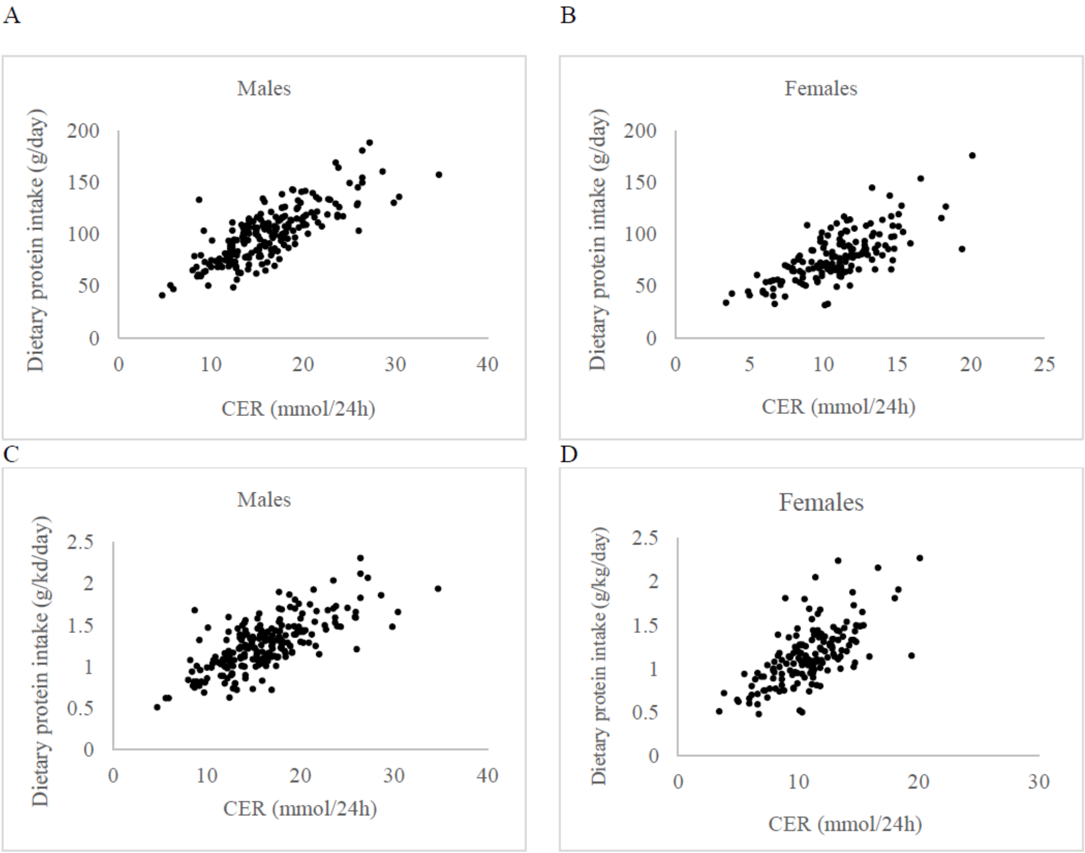

Figure S2. Scatterplots with urinary creatinine excretion on the x-axis and dietary protein intake in gram/day and gram/kg/day on the y-axis, stratified by sex. (A) is dietary protein intake in g/day for males, (B) is dietary protein intake in g/day for females, (C) is dietary protein intake in g/kg/day for males and (D) is dietary protein intake in g/kg/day for females.
